# Rehabilitation for Patients with COVID-19-Associated Acute Respiratory Distress Syndrome During Quarantine: A Single-Center Experience

**DOI:** 10.3390/medicina60101719

**Published:** 2024-10-20

**Authors:** Myung Hun Jang, Yong Beom Shin, Ho Jeong Shin, Eunsuk Jeong, Saerom Kim, Wanho Yoo, Hyojin Jang, Kwangha Lee

**Affiliations:** 1 Department of Rehabilitation Medicine, Pusan National University Hospital, Busan 49241, Republic of Korea; zmh1048@naver.com (M.H.J.); yi0314@gmail.com (Y.B.S.); 2 Biomedical Research Institute, Pusan National University Hospital, Busan 49241, Republic of Korea; kimsaerom03@naver.com (S.K.); lcc2202@naver.com (W.Y.); hyoding2@naver.com (H.J.); 3 Department of Rehabilitation Medicine, Pusan National University School of Medicine, Busan 49241, Republic of Korea; 4 Department of Physical Therapy, Graduate School, Catholic University of Pusan, Busan 49241, Republic of Korea; ptshin7@gmail.com; 5 Division of Pulmonary, Allergy, and Critical Care Medicine, Department of Internal Medicine, Pusan National University Hospital, Busan 49241, Republic of Korea; triniti1024@hanmail.net; 6 Department of Internal Medicine, Pusan National University School of Medicine, Busan 49241, Republic of Korea

**Keywords:** COVID-19, acute respiratory distress syndrome, intensive care unit, mechanical ventilation, rehabilitation

## Abstract

*Background and Objectives*: In this study, we evaluated clinical factors associated with implementing a rehabilitation program for patients with COVID-19-associated acute respiratory distress syndrome (ARDS) requiring mechanical ventilation (MV) during the quarantine period. *Materials and Methods*: This observational study was conducted in the National Designated Isolated ICU, a dedicated COVID-19 center with 18 beds, from 30 December 2020 to 30 May 2022. One hundred and fifty-four patients (mean age: 67.3 ± 13.4 years; male: 59.7%) were enrolled. The ICU rehabilitation program included early mobilization, chest physiotherapy, and dysphagia treatment. *Results*: Forty-five patients (29.3%) participated in the rehabilitation program. Multivariate logistic regression identified three significant factors: tracheostomy (odds ratio [OR], 2.796; 95% confidence interval [CI], 1.238–6.316; *p* = 0.013), body mass index ≥ 25.0 kg/m^2^ (OR, 2.724; 95% CI, 1.276–5.817; *p* = 0.010), and extracorporeal membrane oxygenation (OR, 2.931; 95% CI, 1.165–7.377; *p* = 0.022); patients with all three factors were younger (median 44 vs. 70 years, *p* < 0.001) and had significantly lower Acute Physiology and Chronic Health Evaluation II scores (median 12 vs. 16, *p* = 0.002) on the MV day. One-year cumulative mortality rates for patients with 0 to 3 factors were 66.7%, 50.8%, 38.9%, and 15.4%, respectively, with a significant difference among them (log-rank, *p* < 0.001). *Conclusions*: Three clinical factors associated with implementing a rehabilitation program during the quarantine period for COVID-19-associated ARDS patients were identified. The program was feasible and beneficial, particularly for younger patients with lower illness severity and fewer comorbidities on the day of MV.

## 1. Introduction

During the COVID-19 pandemic, a significant number of hospitalized patients developed acute respiratory distress syndrome (ARDS), which required invasive mechanical ventilation (MV) in intensive care units (ICUs). In such patients, prolonged ventilator support raises the risk of ICU-acquired weakness, which can increase morbidity and mortality [1,2]. To address this, the feasibility and effectiveness of a pulmonary rehabilitation program, as an essential component of managing patients with COVID-19-associated ARDS requiring MV, have been investigated [3,4,5].

Despite the establishment of specialized ICUs for critically ill patients with COVID-19, several challenges remain, including limited critical care resources, constrained tertiary care hospital facilities, and a shortage of critical care personnel [6]. These issues hinder the widespread implementation of rehabilitation programs, and this is further complicated by the lack of standardized guidelines for managing patients with COVID-19-associated ARDS on MV. The unique challenges posed by the quarantine period necessitate reevaluation of rehabilitation strategies. To date, there has been limited research on the implementation of such programs for patients with ARDS requiring MV during the COVID-19 quarantine period in South Korea.

In response to these challenges, the aims of this study were to evaluate the practical implementation of a comprehensive rehabilitation program for patients with COVID-19-associated ARDS admitted to the National Designated Isolated ICU during the quarantine period. The study focused on identifying clinical factors associated with increased participation in rehabilitation programs and assessing their impact on patient outcomes.

## 2. Materials and Methods

### 2.1. Study Design and Patient Selection

In this observational study, patients admitted to the National Designated Isolated ICU, a dedicated COVID-19 center with 18 ICU beds, were investigated from 30 December 2020 to 30 May 2022. This ICU, located in a regional hospital, was established temporarily to treat critically ill patients with COVID-19, offering comprehensive cardiovascular care and continuous airway monitoring. The nurse-to-bed ratio in this respiratory ICU was maintained at 1:1.

The study included adult patients aged ≥ 18 years who required MV following initial endotracheal intubation. Exclusion criteria included patients with irreversible brain injury or neuromuscular diseases. Patients diagnosed with ARDS according to the Berlin definition were included [7]. All patients received management according to lung-protective ventilator strategies [8], and were treated with dexamethasone and/or remdesivir, as previously reported [9,10]. Additionally, all ventilated patients had access to an ICU rehabilitation program, including mobilization, strengthening exercises, chest physiotherapy, and individualized dysphagia treatment, administered by a team comprising a physiatrist, physiotherapist, nurse, and occupational therapist. The quarantine period followed the guidelines, with the duration for each patient determined according to directives issued by the Busan Civil Facilitation Division.

### 2.2. Ethics Approval

The study protocol received approval from the Institutional Review Board (IRB) of the institute (2407-021-141). Given the observational nature of the study, the IRB waived the requirement for informed consent from enrolled patients or their families or surrogates. It is essential to note that this study had no impact on the treatment provided to the enrolled patients.

### 2.3. Data Collection

Demographic and clinical data were retrospectively collected, including age, sex, body mass index (BMI), length of stay (LOS) in the ICU and hospital, duration on MV, and both in-hospital and 1-year mortality rates. Data were extracted from electronic medical records. Severity of illness was assessed using the Acute Physiology and Chronic Health Evaluation (APACHE) II score [11], and organ failure was evaluated using the Sequential Organ Failure Assessment (SOFA) score [12]. Charlson’s comorbidity index was used to assess underlying and pre-existing comorbidities [13]. Inflammatory markers, such as procalcitonin and C-reactive protein, were recorded on the day of MV initiation. Tracheostomy status and septic shock presence on the day of MV were evaluated according to Sepsis-3 definitions [14].

Further, the number of patients who received specific therapies, including hemodialysis, prone positioning, and extracorporeal membrane oxygenation (ECMO), during hospitalization was investigated. Hemodialysis was considered if any form of renal replacement therapy was initiated, either on the day of MV or within 72 h thereafter. Prone positioning followed criteria established by the PROSEVA trial [15,16]. Eligible patients, based on the EOLIA trial criteria [17], received veno-venous ECMO support for life-threatening hypoxemia.

A comprehensive evaluation of patients involved in the rehabilitation program, led by a team of healthcare professionals, was recorded during the quarantine period. The in-hospital mortality rate and the cumulative 1-year mortality rate were assessed. In-hospital mortality was defined as any death occurring before hospital discharge, while 1-year mortality was defined as mortality within 1 year after initiation of MV. One-year mortality was assessed by reviewing the National Health Insurance Service database for surviving patients discharged from the hospital.

### 2.4. Statistical Analysis

Continuous variables following a normal distribution are presented as mean ± standard deviation and were compared using the Student’s t-test. Analysis of variance was conducted to determine the significance of differences among groups. Non-normally distributed continuous variables are presented as median (range) and were compared using the Wilcoxon rank-sum test. Categorical variables are presented as numbers (percentages) and were compared using either the χ^2^ test or Fisher’s exact test, with Fisher’s test used for small sample sizes.

To identify factors associated with a single rehabilitation program implementation during the quarantine period, stepwise logistic regression analysis was conducted. Variables with *p* < 0.10 in univariate analysis were included in the multivariate analysis, with their corresponding 95% confidence intervals. β coefficient values from multivariate logistic regression analysis were simplified to natural numbers > 0 and Kaplan–Meier estimates used to predict 1-year survival, stratified by these factors. Optimal cut-off values for clinical variables to identify program implementation during the quarantine period were established by maximizing Youden’s index [18].

All statistical analyses were two-tailed, with *p* < 0.05 considered statistically significant. SPSS version 27.0 for Windows (SPSS Inc., Chicago, IL, USA) and MedCalc version 22.021 (MedCalc Software, Ostend, Belgium) were used for all analyses.

## 3. Results

### 3.1. Patient Characteristics

**Figure 1:**
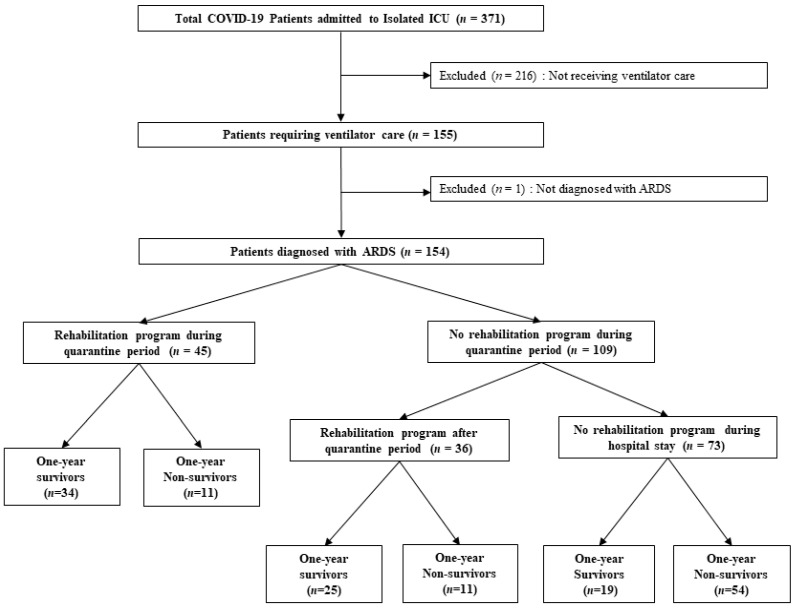
Flow chart showing patient selection and outcomes.

During the study period, 371 patients were admitted to the Isolated ICU, with 154 (41.5%) receiving ventilator care and diagnosed with ARDS (Figure 1). The in-hospital mortality rate for this group was 40.9%, with a 1-year cumulative mortality rate of 49.4%. Comparisons of the clinical characteristics and outcomes between survivors and non-survivors (one-year mortality) are presented in Table 1. Survivors were younger, had a higher BMI, and had longer ICU, hospital, and MV stays than non-survivors. Survivors also had significantly lower APACHE II and SOFA scores, lower Charlson’s comorbidity index, and fewer underlying comorbidities, including cardiovascular disease and chronic kidney disease. Further, survivors had higher rates of hemodialysis within 72 h after MV initiation, were more likely to undergo tracheostomy, and more frequently participated in rehabilitation programs during the quarantine period.

### 3.2. Participation in a Rehabilitation Program During Quarantine

A total of 45 patients (29.3%) participated in the rehabilitation program during the quarantine period, with a median time of 12 days (range 2 to 40 days) between ICU admission and program commencement. Participants were younger, had a higher BMI, and lower APACHE II and SOFA scores than non-participants. They also had lower rates of hemodialysis within 72 h after MV initiation, a higher proportion of ECMO insertions, and were more likely to have undergone tracheostomy during the quarantine period. Participants in the rehabilitation program had lower in-hospital and 1-year cumulative mortality rates than those of non-participants (Table 2).

### 3.3. Clinical Factors Affecting Rehabilitation Program Implementation During Quarantine

**Figure 2:**
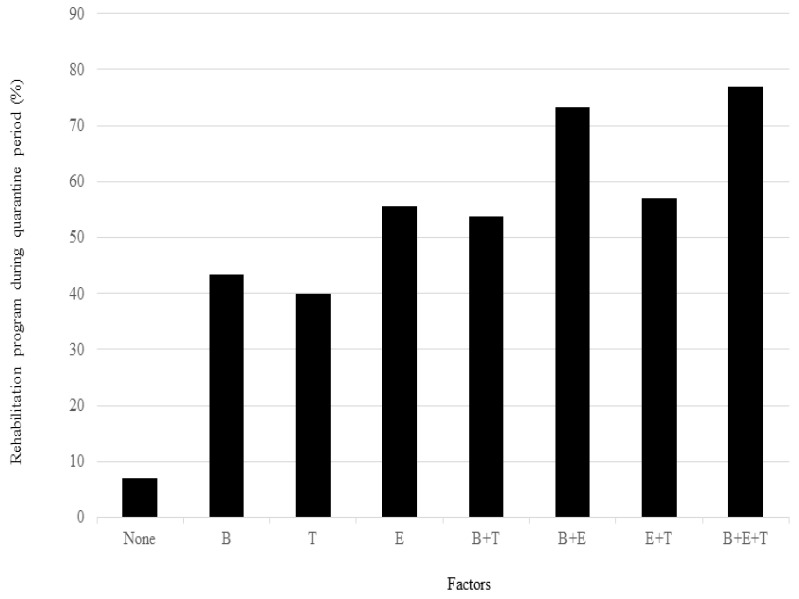
Participation rate in the rehabilitation program according to numbers of factors associated with participation in the program. Abbreviations: B, BMI ≥ 25.0 kg/m^2^; T, tracheostomy during quarantine period; E, ECMO during quarantine period.

Clinical factors associated with the rehabilitation program implementation during the quarantine period, based on analysis using a logistic regression model, are presented in Table 3. A multivariate logistic regression analysis identified three factors significantly associated with rehabilitation program implementation during quarantine: tracheostomy during the quarantine period, BMI ≥ 25.0 kg/m^2^, and a requirement for ECMO. Additionally, the rate of participation in the rehabilitation program increased with the number of these three factors occurring in each patient (Figure 2).

**Figure 3:**
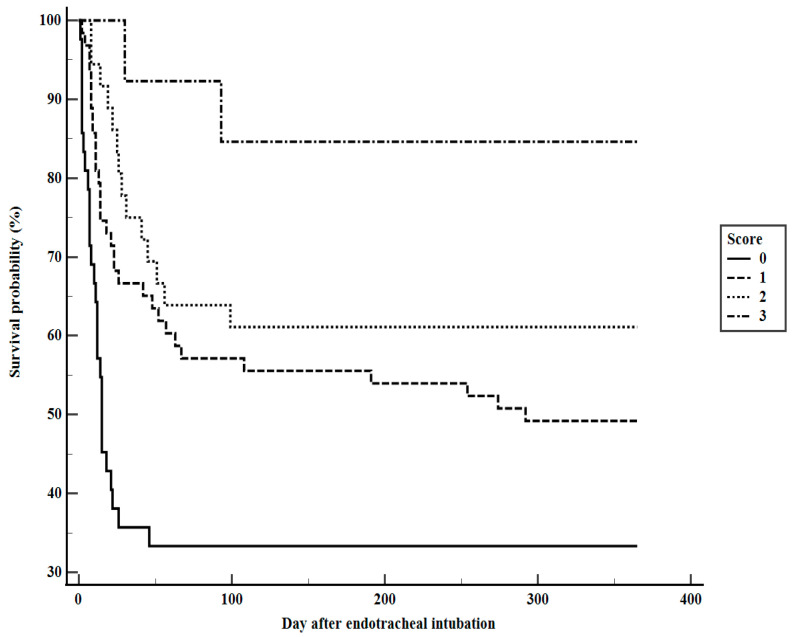
Kaplan–Meier survival curves of patients stratified according to number of factors associated with participation in the rehabilitation program (log-rank *p* < 0.001).

No weights were assigned to these three variables based on the β coefficient values from the multivariate logistic regression analysis (Table 3). In a further comparison of clinical characteristics based on the number of factors associated with rehabilitation program participation, patients with all three factors were younger and had significantly lower APACHE II scores on the MV day, lower Charlson’s comorbidity index, and longer MV and hospital LOS durations (Table 4). One-year cumulative mortality rates for patients with zero to three factors were 66.7%, 50.8%, 38.9%, and 15.4%, respectively, with a significant difference among them (log-rank, *p* < 0.001) (Figure 3).

## 4. Discussion

The aims of this study were to evaluate the practical implementation of a comprehensive rehabilitation program during the quarantine period for patients with COVID-19-associated ARDS admitted to the National Designated Isolated ICU during the COVID-19 pandemic. There are few literature reports on the significance of rehabilitation programs for critically ill patients requiring MV in South Korea; to our knowledge, this is the first study in our country to report the application of a rehabilitation program for patients with ARDS requiring MV during the COVID-19 pandemic quarantine period.

The primary significance of our research lies in identifying specific clinical factors associated with increased participation in a rehabilitation program. Understanding these factors is crucial because it will enable critical care physicians and physiatrists to select patients who are most likely to benefit from early and structured rehabilitation interventions. Tailoring rehabilitation strategies based on these factors can enhance recovery and improve the outcomes of critically ill patients during severe respiratory infection pandemics, such as COVID-19.

Analysis of the enrolled patients revealed that implementation of our rehabilitation program during the quarantine period was more common among those who underwent tracheostomy during the quarantine period, those with a BMI ≥ 25.0 kg/m^2^, and those requiring ECMO. For patients who underwent tracheostomy, rehabilitation program implementation was facilitated by several advantages, including enhanced patient comfort, reduced reliance on sedative agents, accelerated MV weaning, and a lower incidence of nosocomial pneumonia [19,20]. Tracheostomized patients with lower inspiratory pressure and oxygen requirements could potentially be managed with a home ventilator on the general ward, thereby offering a potential solution to reduced ICU capacity during the pandemic [21]. Consequently, the decision of physicians to refer patients to the rehabilitation program may have been influenced by the positive impact of tracheostomy. Interestingly, the rehabilitation program was also more frequently implemented in groups for which rehabilitation is typically challenging, such as those with obesity and/or undergoing ECMO for life-threatening hypoxemia. Further analysis showed that these patients were younger and had less severe illness and comorbidities at ICU admission. Hence, our findings indicate that even patients for whom rehabilitation was considered challenging during the quarantine period could benefit from such programs if they are younger and have less severe illness. Thus, our data suggest that consideration of rehabilitation programs during quarantine periods for patients meeting these parameters is warranted, despite various challenges.

Our findings indicate that introduction of rehabilitation programs for severely ill patients during the COVID-19 outbreak was important. An international recommendation for a protocolized rehabilitation approach, focusing on early mobilization and ventilator liberation, has been published [22]; however, there is currently no national guideline regarding the use of this program for critically ill patients, regardless of whether or not a severe respiratory infectious disease pandemic is underway. Additionally, the Korean government does not cover medical expenses related to ICU rehabilitation. Recent studies in South Korea demonstrated that ICU rehabilitation may contribute to improved survival of critically ill patients [23,24], emphasizing the need for formal coverage of rehabilitation-related medical expenses for such patients.

There are limitations to our study. First, our findings underscore the importance of considering specific clinical factors to effectively target patients who are most likely to benefit from rehabilitation programs during a quarantine period. We were unable to identify additional clinical factors due to the small number of enrolled patients. Second, we did not account for potential variations in rehabilitation program implementation across different ICUs. Future research should focus on multicenter trials to validate these findings and explore standardized rehabilitation protocols that can be universally applied. Lastly, the retrospective, observational design and single-center setting of our study may limit the generalizability of the results. Future research should include larger, multicenter trials to confirm these findings and further investigate the outcomes of patients participating in rehabilitation programs, providing deeper insights into their overall efficacy and potential for broader application in critical care settings.

## 5. Conclusions

In conclusion, our study demonstrates that the implementation of a comprehensive rehabilitation program during the quarantine period for patients with COVID-19-associated ARDS is feasible and beneficial, particularly for those who are younger and have less severe illness. Identifying specific clinical factors, such as tracheostomy, higher BMI, and ECMO requirements, can help critical care physicians target patients who are most likely to benefit from rehabilitation interventions. Despite the limitations of our study, including its retrospective design and single-center setting, the findings provide valuable insights into optimizing critical care and resource allocation during pandemics. Future multicenter trials are necessary to validate these results and develop standardized rehabilitation protocols to enhance patient outcomes in diverse ICU settings.

## Figures and Tables

**Table 1:** Patient clinical characteristics.

Variable	Total (n = 154)	One-Year Survivors (n = 78)	One-Year Non-Survivors (n = 76)	*p*-Value
Male, n (%)	92 (59.7)	41 (52.6)	51 (67.1)	0.073
Age (years)	67.3 ± 13.4	61.8 ± 14.7	73.0 ± 9.0	<0.001
BMI (kg/m^2^) ^(a)^	24.5 ± 4.1	25.6 ± 4.2	23.3 ± 3.8	<0.001
ICU LOS (days)	31 (2–139)	42 (12–139)	17 (2–108)	<0.001
MV LOS (days)	15 (1–108)	18 (5–98)	14 (1–108)	0.042
Hospital LOS (days)	31 (2–139)	42 (13–139)	17 (2–108)	<0.001
APACHE II score ^(a)^	17.0 ± 5.8	15.1 ± 4.8	19.0 ± 5.9	<0.001
SOFA score ^(a)^	6.8 ± 3.1	6.0 ± 2.6	7.6 ± 3.3	0.001
Charlson’s comorbidity index	2.2 ± 1.8	1.6 ± 1.5	2.7 ± 2.0	<0.001
Most common comorbidities before admission				
Diabetes	62 (40.3)	27 (34.6)	35 (46.1)	0.189
Chronic neurologic diseases ^(b)^	45 (29.2)	18 (23.1)	27 (35.5)	0.111
Cardiovascular diseases	26 (16.9)	6 (7.7)	20 (26.3)	0.002
Hemato-oncologic diseases	25 (16.2)	10 (12.8)	15 (19.7)	0.279
Chronic kidney diseases	22 (14.3)	3 (3.8)	19 (25.0)	<0.001
Chronic lung diseases ^(c)^	15 (9.7)	6 (7.7)	9 (11.8)	0.426
ARDS severity				
Mild	28 (18.2)	15 (19.2)	13 (17.1)	0.880
Moderate	82 (53.2)	40 (51.3)	42 (55.3)	
Severe	44 (28.6)	23 (29.5)	21 (27.6)	
Septic shock	35 (22.7)	10 (12.8)	25 (32.9)	0.004
NMBAs during first 48 h after endotracheal intubation	147 (95.5)	75 (96.2)	72 (94.7)	0.717
Prone position during invasive MV	48 (31.2)	30 (38.5)	18 (23.7)	0.056
ECMO insertion during hospital stay ^(d)^	27 (17.5)	17 (21.8)	10 (13.2)	0.204
Hemodialysis within 72 h after MV ^(e)^	13 (8.4)	1 (1.3)	12 (15.8)	0.001
Tracheostomy during quarantine period	85 (55.2)	53 (67.9)	32 (42.1)	0.002
Rehabilitation program during COVID-19 quarantine ^(f)^	45 (29.2)	34 (43.6)	11 (14.5)	<0.001

Normally distributed continuous variables are reported as the mean ± standard deviation (SD); non-normally distributed continuous variables are reported as the median (range); and categorical variables are reported as number (%). (a) All clinical data were calculated or obtained from medical records on the day of ICU admission. (b) Chronic neurologic diseases included cerebrovascular accidents, intracerebral hemorrhage, subdural hemorrhage, subarachnoid hemorrhage, and Alzheimer’s dementia. (c) Chronic lung diseases included chronic obstructive pulmonary disease, interstitial lung disease, and lung collapse due to various causes. (d) All cases received veno-venous ECMO. (e) The need for hemodialysis depended on whether any form of renal replacement therapy was initiated either on the day of MV or within 72 h thereafter. (f) The rehabilitation program included mobilization, strengthening exercises, and chest physiotherapy provided by the ICU rehabilitation team. Abbreviations: BMI, body mass index; ICU, intensive care unit; LOS, length of stay; MV, mechanical ventilation; APACHE II, Acute Physiology and Chronic Health Evaluation II; SOFA, Sequential Organ Failure Assessment; ARDS, acute respiratory distress syndrome; NMBA, neuromuscular blocking agent; ECMO, extracorporeal membrane oxygenation; COVID-19, coronavirus disease 19.

**Table 2:** Comparison of patients with and without rehabilitation program during quarantine.

Variable	Rehabilitation Program During Quarantine ^(a)^		*p*-Value
	Yes (n = 45)	No (n = 109)	
Male	27 (60.0)	65 (59.6)	>0.999
Age, years	66 (27–86)	72 (19–88)	0.012
BMI, kg/m^2^	26.2 ± 4.3	23.8 ± 3.9	0.001
APACHE II score ^(b)^	15.1 ± 5.1	17.8 ± 5.8	0.007
SOFA score ^(b)^	6.4 ± 2.8	7.0 ± 3.1	0.319
ICU LOS	45 (7–139)	25 (2–108)	<0.001
MV LOS	22 (7–85)	14 (1–108)	0.007
Hospital LOS	45 (7–139)	26 (2–108)	<0.001
Charlson’s comorbidity index	1.9 ± 1.9	2.3 ± 1.8	0.227
Most common comorbidities before admission			
Diabetes	17 (27.4)	45 (41.3)	0.721
Chronic neurologic diseases ^(c)^	8 (17.8)	37 (33.9)	0.052
Cardiovascular diseases	7 (15.6)	19 (17.4)	>0.999
Hemato-oncologic diseases	6 (13.3)	19 (17.4)	0.635
Chronic kidney diseases	5 (11.1)	17 (15.6)	0.615
Chronic lung diseases ^(d)^	6 (13.3)	9 (8.3)	0.374
ARDS severity: severe	22 (48.9)	22 (20.1)	0.001
Hemodialysis within 72 h after MV ^(e)^	0 (0.0)	13 (11.9)	0.011
Septic shock on the day of MV	6 (13.3)	29 (26.6)	0.092
Inflammatory markers on the day of MV			
C-reactive protein (mg/dL)	10.5 (0.8–44.2)	10.8 (0.1–38.1)	0.729
Procalcitonin (ng/mL)	0.2 (0.1–10.9)	0.3 (0.1–67.2)	0.178
Prone position during quarantine period	17 (37.8)	31 (28.4)	0.259
ECMO insertion during quarantine period	15 (33.3)	12 (11.0)	0.002
Tracheostomy during quarantine period	34 (75.6)	51 (46.8)	0.001
In-hospital mortality	8 (17.8)	55 (50.5)	<0.001
One-year cumulative mortality	11 (24.4)	65 (59.6)	<0.001

Normally distributed continuous variables are reported as the mean ± standard deviation (SD); non-normally distributed continuous variables are reported as the median (range); and categorical variables are reported as number (%). (a) The rehabilitation program included mobilization, strengthening exercises, and chest physiotherapy provided by the ICU rehabilitation team. (b) All clinical data were calculated or obtained from medical records on the day of ICU admission. (c) Chronic neurologic diseases included cerebrovascular accidents, intracerebral hemorrhage, subdural hemorrhage, subarachnoid hemorrhage, and Alzheimer’s dementia. (d) Chronic lung diseases included chronic obstructive pulmonary disease, interstitial lung disease, and lung collapse due to various causes. (e) The need for hemodialysis was dependent on whether any form of renal replacement therapy was initiated either on the day of MV or within 72 h thereafter. Abbreviations: BMI, body mass index; APACHE II, Acute Physiology and Chronic Health Evaluation II; SOFA, Sequential Organ Failure Assessment; ICU, intensive care unit; LOS, length of stay; ARDS, acute respiratory distress syndrome; ECMO, extracorporeal membrane oxygenation; MV, mechanical ventilation.

**Table 3:** Logistic regression analysis of factors associated with rehabilitation program ^(a)^ implementation during quarantine.

Variable	Univariate OR (95% CI)	*p*-Value	Multivariate OR (95% CI)	*p*-Value	β-Coefficient
Tracheostomy during quarantine	3.515 (1.616–7.645)	0.002	2.796 (1.238–6.316)	0.013	1.028
BMI ≥ 25.0 kg/m^2 (b)^	3.171 (1.545–6.511)	0.002	2.724 (1.276–5.817)	0.010	1.002
Requirement for ECMO during quarantine	4.042 (1.706–9.575)	0.002	2.931 (1.165–7.377)	0.022	1.075
APACHE II score ≤ 16 ^(c)^	3.106 (1.533–6.292)	0.023			
Without septic shock on day of MV	2.356 (0.903–6.146)	0.080			

All variables were included in the multivariate analysis using stepwise backward selection procedures (Hosmer–Lemeshow chi-square = 4.962, df = 5, *p*-value = 0.421). (a) The rehabilitation program included mobilization, strengthening exercises, and chest physiotherapy provided by the ICU rehabilitation team. (b) Cut-off value based on the maximum Youden’s index (AUC, 0.667; 95% CI, 0.586–0.740; *p* = 0.001; sensitivity, 64.4%; specificity, 64.2%). (c) APACHE II score was calculated from medical records on the day of ICU admission. Cut-off value is based on the maximum Youden’s index (AUC, 0.632; 95% CI, 0.551–0.708; *p* = 0.008; sensitivity, 68.9%; specificity, 51.4%). Abbreviations: OR, odds ratio; CI, confidence interval; BMI, body mass index; ECMO, extracorporeal membrane oxygenation; APACHE II, Acute Physiology and Chronic Health Evaluation II; MV, mechanical ventilation; AUC, area under the receiver operating characteristic curve.

**Table 4:** Comparison of clinical characteristics based on numbers of factors associated with participation in a rehabilitation program ^(a)^.

	Number of Factors = 0 (n = 42)	Number of Factors = 1 (n = 63)	Number of Factors = 2 (n = 36)	Number of Factors = 3 (n = 13)
Male	30 (71.4)	39 (61.9)	15 (41.7)	8 (61.5)
Age (years)	68.1 ± 12.2 ^(c)^	71.8 ± 9.4 ^(c)^	66.6 ± 11.6 ^(c)^	45.0 ± 16.4 ^(d)^
APACHE II score ^(b)^	18.5 ± 5.9 ^(c)^	17.8 ± 6.0 ^(c)^	15.6 ± 4.1 ^(c)^	12.4 ± 4.5 ^(d)^
SOFA score ^(b)^	7.3 ± 3.4	7.0 ± 3.1	6.2 ± 2.3	5.9 ± 2.9
Charlson’s comorbidity index	2.4 ± 2.0 ^(c)^	2.4 ± 1.9 ^(c)^	2.0 ± 1.5 ^(c)^	1.2 ± 2.0 ^(d)^
MV LOS	10 (1–33) ^(c)^	15 (2–108) ^(d)^	22 (8–85) ^(d)^	30 (9–98) ^(e)^
Hospital LOS	18 (2–92) ^(c)^	28 (3–139) ^(d)^	45 (8–117) ^(d)^	48 (20–98) ^(e)^

Normally distributed continuous variables are reported as the mean ± standard deviation (SD); non-normally distributed continuous variables are reported as the median (range); and categorical variables are reported as number (%). (a) The three factors (BMI ≥ 25.0 kg/m^2^, requirement of ECMO, and tracheostomy during quarantine period) were those significantly associated with rehabilitation program implementation in multivariate logistic regression analysis. (b) APACHE II and SOFA scores were calculated from medical records on the day of ICU admission. (c–e) Significant difference; ANOVA *p* < 0.005. Abbreviations: BMI, body mass index; APACHE II, Acute Physiology and Chronic Health Evaluation II; SOFA, Sequential Organ Failure Assessment; ECMO, extracorporeal membrane oxygenation; MV, mechanical ventilation; LOS, length of stay; ICU, intensive care unit; ANOVA, analysis of variance.

## Data Availability

The data presented in this study are available upon request from the corresponding author.
